# Effect of Cooling and Freezing on Llama (*Lama glama*) Sperm Ultrastructure

**DOI:** 10.3389/fvets.2020.587596

**Published:** 2020-10-28

**Authors:** Renato Zampini, Ximena A. Castro-González, Luciana M. Sari, Alfredo Martin, Ana V. Diaz, Martin E. Argañaraz, Silvana A. Apichela

**Affiliations:** ^1^Instituto Superior de Investigaciones Biológicas (INSIBIO), Consejo Nacional de Investigaciones Científicas y Técnicas-Universidad Nacional de Tucumán (CONICET-UNT), Instituto de Biología “Dr. Francisco D. Barbieri”, Facultad de Bioquímica, Química y Farmacia, Universidad Nacional de Tucumán (UNT), San Miguel de Tucumán, Argentina; ^2^Cátedra de Biología Celular y Molecular, Facultad de Bioquímica, Química y Farmacia, Universidad Nacional de Tucumán (UNT), San Miguel de Tucumán, Argentina; ^3^Cátedra de Técnicas Quirúrgicas, Facultad de Agronomía y Zootecnia, Universidad Nacional de Tucumán (UNT), San Miguel de Tucumán, Argentina; ^4^Instituto de Investigación Animal del Chaco Semiárido (IIACS), Instituto Nacional de Tecnología Agropecuaria (INTA), Leales, San Miguel de Tucumán, Argentina; ^5^Cátedra de Zootecnia General I, Facultad de Agronomía y Zootecnia, Universidad Nacional de Tucumán (UNT), San Miguel de Tucumán, Argentina

**Keywords:** cryodamage, cryopreservation, *Lama glama*, ultrastructure, South American camelids, spermatozoa

## Abstract

Semen cryopreservation in South American camelids has a low efficiency. Post-thaw viability of sperm is low, and poor results are obtained when artificial insemination is performed with cryopreserved semen, impeding advances both in accelerated genetic progress and selection. This study aimed to describe the effect of a conventional method of camelid semen cryopreservation on the llama sperm ultrastructure during cooling and freezing, using transmission and scanning electron microscopy (TEM, SEM). Sperm motility, vigor, viability, and DNA integrity during those steps were also examined. Ejaculates from five fertile adult llama males were obtained by electroejaculation. For cooling, semen samples were washed with Hepes-balanced salt solution (HBSS), diluted in Tris-citric acid-fructose egg yolk extender (TCF-EY), and then cooled until 5°C for 24 h. For freezing, sperm samples were washed with HBSS, diluted in TCF-EY and cooled until 5°C for 2.5 h. Samples were equilibrated with TCF-EY, supplemented with 6% glycerol at 5°C for 20 min, and then stored in liquid nitrogen for a month before thawing. TEM and SEM analyses were carried out on sperm samples prior to cryopreservation, after cooling down until 5°C for 2.5 and 24 h, and after the freeze-thaw process. Ultrastructural injury was noticed during cooling, even though sperm motility, vigor, viability, and DNA integrity were not significantly affected. Analysis revealed plasma membrane and acrosome damage, loss of mitochondria, and axoneme and periaxonemal structure disorganization after 2.5 h of cooling. During freezing, a significant decrease in sperm motility and viability was observed after thawing. TEM and SEM revealed prominent signs of post-thawing damage. The plasma membrane was lost or exhibited various degrees of swelling, undulation, and perforations. Besides, the sperm presented vacuoles in the nucleus and broken acrosomes. Mitochondria in the midpiece showed vacuolization and structural disorganization. In conclusion, SEM and TEM revealed that cryopreservation induced ultrastructural damages in llama sperm that initiated during cooling and intensified during freezing. These details provide valuable data for further studies to minimize cryodamage in camelid sperm.

## Introduction

Semen cryopreservation is a widely used technique to preserve and supply sperm for breeding and maintenance of genetic diversity in wildlife. During a standard cryopreservation protocol, different processing steps are involved (dilution of semen at 37°C with the extender and cooling until 5°C, addition of a cryoprotectant and equilibration, and freezing in liquid nitrogen at −196°C), and it is not entirely clear how each step affects the sperm cell (1). However, it has been shown that cryopreservation induces deleterious alterations in sperm structure and function (2–5). These involve thermal stress due to the change in temperature during cooling, freezing and thawing, as well as osmotic stress caused by the addition of high concentrations of cryoprotective agents and crystallization (6). As a result, a reduction in overall sperm fertility has been reported in different domestic livestock when performing artificial insemination (AI) (7, 8).

In South American camelids, poor results have been obtained after AI with cryopreserved semen (9–14). Llama and alpaca pregnancy rates with frozen-thawed semen ranged from 0 to 26% (9–12), while maximum pregnancy (33%) was obtained with cooled semen at 5°C (14).

Ultrastructural damage after cryopreservation has been reported in bull (1), goat (15), dromedary (16), ram (17), dog (18), and human (19), many of which cannot be detected by conventional assessment. Ultrastructural analysis requires high magnification, which is not possible with a light microscope due to the low resolution. Electron microscopy is an extremely useful tool for this purpose. Specifically, scanning electron microscopy (SEM) offers a three-dimensional image of surface structures and transmission electron microscopy (TEM) provides a high magnification image of cellular components, including the cytoskeleton, membrane systems, organelles, as well as specialized structures in differentiated cells.

To date, no studies have examined sperm ultrastructure alterations in llamas caused by cooling or freezing. Deeper knowledge about these procedures could be useful to improve the most critical steps during cryopreservation. Therefore, the present study aimed to evaluate the effects of cryopreservation on the ultrastructural characteristics of llama sperm during cooling until 5°C and freezing. Standard assessment of sperm motility, vigor, viability and DNA integrity was also performed.

## Materials and Methods

### Animals

Five llama males between 4 and 5 years old from the CEEC (Centro Experimental de Estudios en Camélidos Sudamericanos), Faculty of Agronomy and Zootechnics of the National University of Tucumán (26°50′11.4“S 65°16′58.3”W, and 440 m altitude, Tucumán, Argentina) were used. The animals were kept on natural pasture and strategically supplemented with bales of alfalfa, and water was provided *ad libitum*. Semen was obtained during the winter-spring of 2019.

### Semen Collection and Handling

Semen collections were carried out using electroejaculation (EE) under general anesthesia with 0.2 mg/kg of xylazine IV (Xilazina, Richmond, Argentina) and 1.5 mg/kg of ketamine IV (Ketamina, Holliday, Argentina). The same males were subjected to EE with an interval of 15 days between successive semen collections. All procedures were in line with the UNT 002/18 Protocol approved by the Committee for the Care and Use of Laboratory Animals (CICUAL) from the Universidad Nacional de Tucumán, Argentina.

A stimulator similar to an Electrojac V (Sistel, Argentina) with a rectal probe with three linear electrodes was used for EE. The probe was lubricated, gently inserted into the rectum and orientated so that the electrodes were positioned ventrally to the prostate. The device was used in automatic mode, applying stimulus cycles of 2 s with 2 s intervals between stimuli. Voltage was increased one volt every five cycles (starting with 2 V), until erection occurred (~8 V). Then, voltage was increased with 1 V increments every 10 cycles until ejaculation. According to the sensitivity of each animal to electro-stimuli, the minimum voltage required to obtain ejaculation was applied (maximum 13 V), without exceeding 10 min of electro-stimulation.

Semen was obtained in 50 ml Falcon tubes and immediately placed in a 37°C water bath. At this time, an aliquot of 12 μl was used to evaluate sperm viability, motility, and vigor in raw semen. In order to remove seminal plasma, each ejaculate was diluted 4-fold with Hepes balanced salt solution (HBSS: 25 mM Hepes, 130 mM NaCl, 5 mM KCl, 0.36 mM NaH_2_PO_4_, 0.49 mM MgCl_2_, and 2.4 mM CaCl_2_, pH 7.4, 290 mOsm/kg) and subsequently centrifuged at 800 × g for 8 min at room temperature. Particularly highly viscous semen was first diluted and then gently pipetted to break down the gel before centrifuging. The sperm pellet was suspended in 200–300 μl of HBSS, and its concentration was calculated using a Makler counting chamber. At this point, some samples were used for cooling (C0: fresh sperm before cooling procedure) and others for freezing (F0: fresh sperm before freezing procedure) protocols.

### Sperm Cryopreservation

**Figure 1:**
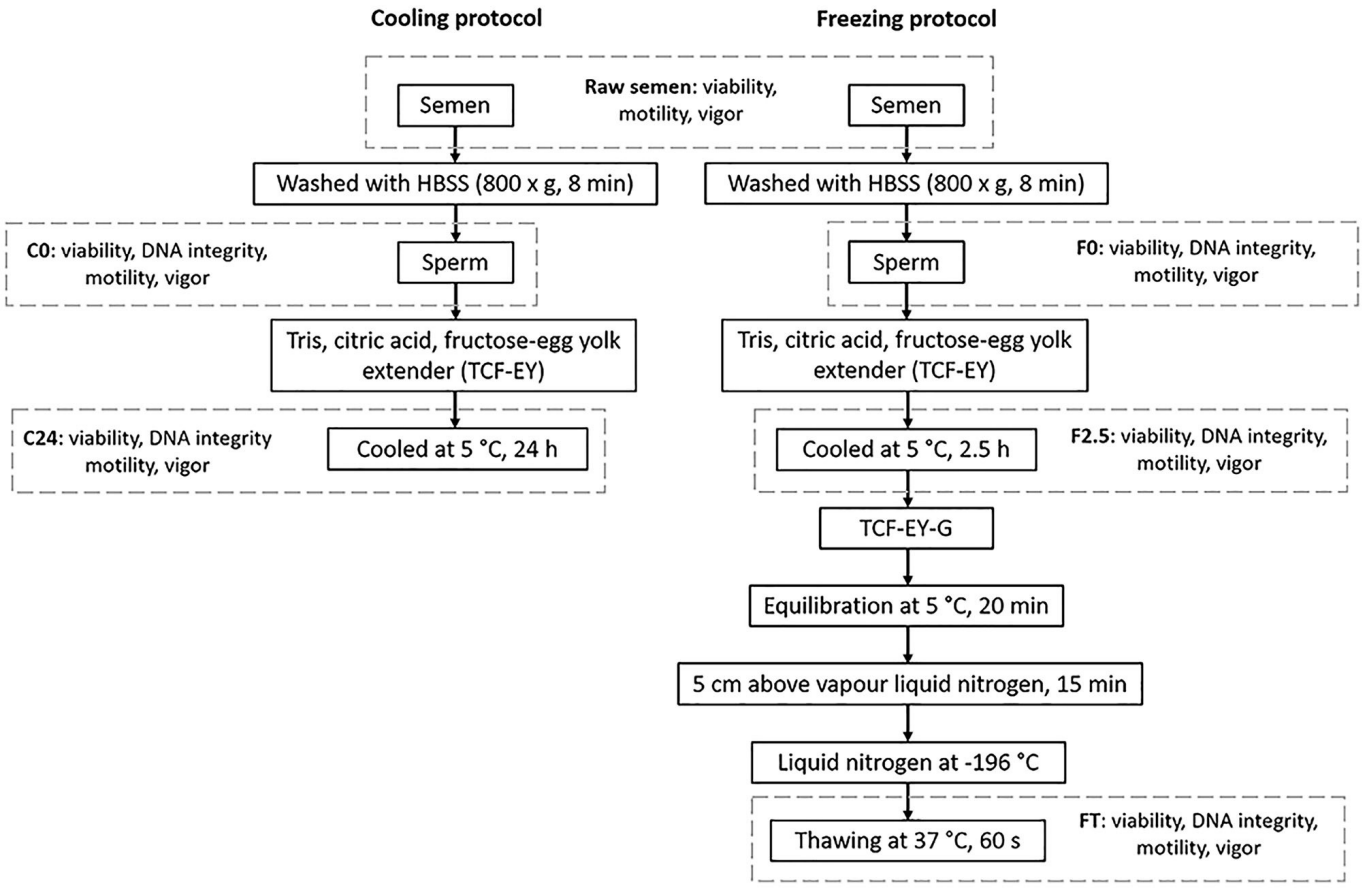
Experimental design of cryopreservation procedures for llama sperm.

Sperm samples were cryopreserved by cooling down until 5°C for 24 h or freezing in liquid nitrogen at −196°C for a month. The experimental design is shown in Figure 1.

#### Cooling Protocol

Sperm suspensions (*n* = 5, *r* = 2) were diluted with Tris citric acid fructose-egg yolk extender (TCF-EY: 250 mM Tris, 80 mM citric acid, 60 mM fructose, 20% egg yolk, 0.5% Equex, 80,000 IU Penicillin G sodium, and 0.1% Streptomycin sulfate) to obtain a final concentration of 30–40 × 10^6^ spermatozoa/ml. Samples were subsequently placed in a 37°C water bath and then put in a refrigerator. The temperature was monitored until reaching 5°C in ± 2.5 h (cooling rate 0.2°C/min). Samples were then maintained at 5°C for 24 h. After this period, the cooled sperm cells were warmed up to 37°C in a water bath (C24) to carry out sperm evaluations.

#### Freezing Protocol

Sperm suspensions (*n* = 5, *r* = 3) were diluted with TCF-EY until a final concentration of 120–160 × 10^6^ spermatozoa/ml, and subsequently cooled down until 5°C for ± 2.5 h (cooling rate 0.2°C/min) (F2.5). Then, sperm cells were diluted 1:1 with freezing diluent that consisted of TCF-EY supplemented with 12% (v/v) glycerol as cryoprotectant (TCF-EY-G) (final glycerol concentration was 6%) followed by equilibration for 20 min at 5°C. After the equilibration period, the samples were loaded in 0.5 ml straws (30–40 × 10^6^ spermatozoa per straw), which were placed 5 cm above liquid nitrogen vapor for 15 min in a freezing unit for straws (Minitube, Madison, WI, USA). Straws were then plunged into liquid nitrogen at −196°C for storage. After a month the samples were thawed in a 37°C water bath for 60 s (FT: freeze-thawing) and evaluated as follows.

### Sperm Features Assessment

The following sperm traits were evaluated: viability, motility, vigor and DNA integrity. The traits were assessed at different time points: C0 and C24 for cooling; and F0, F2.5, and FT for freezing. Additionally, viability, motility, and vigor were also assessed in raw semen.

The percentage of live spermatozoa was determined by the eosin-nigrosin staining. Briefly, 5 μl of sperm suspension was placed on a slide and mixed with the same volume of eosin-nigrosin solution. After 30–40 s, a thin smear was prepared and observed under a light microscope at 400X magnification. At least 200 spermatozoa from two different slides were counted per sample (viable sperm remained colorless, while non-viable sperm stained pink).

Assessments of motility and vigor were made by placing a 7 μl aliquot of spermatozoa on a slide under heating stage and coved with a warmed coverslip (18 × 18 mm), using a bright-field microscope (Numak, model Zenith DO-1L, Buenos Aires, Argentina) at 400X. The patterns observed were: oscillatory motility (OM) and progressive motility (PM). In addition, total sperm motility (TM = OM + PM) was determined. Sperm vigor was evaluated by using a score from 0 to 5 following Table 1 criteria, considering the amount of sperm with movement, the presence of progressive movement, and the beat frequency of the sperm.

**Table 1:** Sperm vigor classification.

**Score**	**Description**
5	Sperm have very fast oscillatory or progressive movement. 60% of cells are motile
4	Sperm have vigorous oscillatory movement, with fast beat frequency. About 40–50% of cells are motile
3	Sperm have mainly oscillatory movement, with slow beat frequency, and low progressive movement. Less than 40% of cells are motile
2	Sperm have weak oscillatory movement. Progressive motility is not observed
1	Total motility is poor. Very few sperm (about 10%), have weak movements
0	Sperm have no movement

To assess sperm DNA integrity, acridine orange assay was carried out on sperm samples. Briefly, the sperm were diluted with HBSS and centrifuged at 800 × g for 8 min to remove the extender, suspended in 100 μl of HBSS (except for C0 and F0 because they have already been washed with HBSS). Then, thin smears were prepared from the sperm suspensions, fixed for 3 h in Carnoy's solution (methanol/acetic acid, 3:1) and stained with acridine orange solution (0.19% in phosphate citrate buffer, pH = 2.5) for 5 min. The slides were gently washed with distilled water for 5 min and air-dried. The stained smears were then observed under a fluorescence microscope (wavelengths of 450–490 nm, Olympus, Tokyo, Japan) at 400X magnification. Sperm with normal DNA content present a green fluorescence, whereas sperm with abnormal DNA content emit fluorescence in a spectrum varying from yellow-green to red. At least 100 spermatozoa per slide were counted.

### Ultrastructural Assessment

Sperm ultrastructure analysis was performed by scanning and transmission electron microscopy at the Centro Integral de Microscopía Electrónica (CIME), CONICET, Tucumán-Argentina.

#### Scanning Electron Microscopy (SEM)

SEM analysis was carried out on sperm samples obtained before cryopreservation (fresh sperm), after cooling at 5°C for 2.5 and 24 h, and after freeze-thawing. Sperm samples (concentration≥ 80 × 10^6^ spermatozoa/ml, *n* = 2 per each group) were washed with 1 ml of HBSS and centrifuged twice at 800 × g for 5 min. The supernatant was removed and the pellets formed by spermatozoa were fixed in Karnovsky's reagent (paraformaldehyde 2.7% and glutaraldehyde 1.7% in sodium phosphate buffer, pH 7.2) for 24 h at 4°C. Then, 100 μl of sperm suspension was placed on a glass previously covered with 2% agar for 1 h. After that, the samples were dehydrated in increasing ethanol concentrations (30, 50, 70, 90, and 100%) for 10 min each, and subjected to two acetone baths for 10 min. The samples were critical point dried with liquid carbon dioxide (Denton Vacuum DCP-1 Critical Point Dryer, NJ, USA), mounted on aluminum stubs and metalized with gold (JEOL ion sputter JFC-1100, Tokyo, Japan) for later observation using a scanning electron microscope (Zeiss SUPRA 55VP, Oberkochen, Germany). Different fields were randomly chosen, and 100 sperm per sample were examined.

#### Transmission Electron Microscopy (TEM)

TEM analysis was carried out on fresh sperm, after cooling at 5°C for 2.5 and 24 h, and after freeze-thawing. Similar to SEM technique, the sperm samples (concentration≥ 80 × 10^6^ spermatozoa/ml, *n* = 2 per each group) were washed with 1 ml of HBSS and centrifuged twice at 800 × g for 5 min. The sperm pellets were fixed in Karnovsky's reagent for 24 h at 4°C. Then, sperm were centrifuged and the pellets were embedded in 1.2% agar solution. Afterward, the samples were washed three times with sodium phosphate buffer, post-fixed in 2% osmium tetroxide in the same buffer at 4°C overnight, and treated with an aqueous solution of 2% uranyl acetate for 30 min. Samples were serially dehydrated in ethanol, passed through acetone and embedded in Spurr resin overnight at 60°C. Ultrafine sections (60–70 nm) were examined on copper grids using a Zeiss Libra 120 electron microscope (Carl Zeiss, Oberkochen, Germany). Micrographs were examined focusing on the different sperm structures (plasma membrane, acrosome, nucleus, mitochondria in the midpiece, and axoneme and periaxonemal in the principal piece), and observations were made paying particular attention to whether the structures were preserved or presented some type of alteration (100 sperm heads and tails per sample including longitudinal and cross-sections were examined) to describe the sperm quality features in the different groups.

### Statistical Analysis

All data were expressed as the mean ± standard error of the mean, and analyzed by statistical packages Infostat Version 2011p and R 3.1. Linear mixed-effects models were used to evaluate the effect of cryopreservation on sperm motility, viability, and DNA integrity, with animals as a random effect, and sampling time as fixed effects. Mean comparisons were performed using Fisher's LSD test. The same effects on vigor were evaluated using the Kruskal–Wallis test. *p* < 0.05 was considered statistically significant.

## Results

### Effect of Cryopreservation on Sperm Traits

Raw semen presented 64.6 ± 3.2% live sperm and 47.0 ± 4.3% total motility; most of the sperm showed oscillatory movement (41.0 ± 4.6%), but very little progressive movement was observed (6.0 ± 2.6%). Mean sperm vigor was 2.7 ± 0.3 vigor score.

Concerning sperm motility (total, progressive, and oscillatory), vigor, viability, and DNA integrity, no differences were found between C0 and C24 sperm samples during cooling (Tables 2, 3).

**Table 2:** Percentage of llama sperm viability, and DNA integrity during cooling procedure.

	**Viability (%)**	**DNA integrity (%)**
C0	67.4 ± 3.8^a^	86.2 ± 4.5^a^
C24	72.7 ± 3.7^a^	82.4 ± 8.6^a^

*Values are mean ± standard error of the mean*.

a *The same letter within columns indicates no significant difference (p > 0.05)*.

**Table 3:** Percentage of llama sperm motility (TM, total motility; PM, progressive motility, and OM, oscillatory movement) and vigor (scale 0–5) during cooling procedure.

	**TM (%)**	**PM (%)**	**OM (%)**	**Vigor (vigor score)**
C0	51.5 ± 6.2^a^	11.0 ± 6.6^a^	40.3 ± 7.4^a^	4.0 ± 0.4^a^
C24	58.3 ± 5.7^a^	24.2 ± 6.0^a^	34.0 ± 6.8^a^	3.6 ± 0.4^a^

*Values are mean ± standard error of the mean*.

a *The same letter within columns indicates no significant difference (p > 0.05)*.

The freezing procedure resulted in a significant reduction in spermatozoa viability after thawing (FT) compared with F0 and F2.5 values (*p* < 0.05), but no differences were detected regarding DNA integrity (Table 4). A marked decrease in oscillatory and total motility was observed after thawing (FT) when compared to F0 and F2.5 sperm samples. Particularly, progressive motility of F2.5 samples was higher than FT ones (Table 5). Sperm vigor was not affected during the freezing protocol (Table 5).

**Table 4:** Percentage of llama sperm viability, and DNA integrity during freezing procedure.

	**Viability (%)**	**DNA integrity (%)**
F0	66.9 ± 2.4^a^	88.7 ± 4.5^a^
F2.5	66.2 ± 2.4^a^	88.0 ± 4.5^a^
FT	36.7 ± 2.4^b^	90.4 ± 4.4^a^

*Values are mean ± standard error of the mean*.

a, b *Within columns, different letters between rows indicate significant differences for each of the sperm characteristic (p < 0.05)*.

**Table 5:** Percentage of llama sperm motility (TM, total motility, PM, progressive motility, and OM, oscillatory movement) and vigor (scale 0–5) during freezing procedure.

	**TM (%)**	**PM (%)**	**OM (%)**	**Vigor (vigor score)**
F0	41.0 ± 4.11^a^	17.3 ± 3.6^ab^	23.7 ± 3.6^a^	3.2 ± 0.3^a^
F2.5	47.0 ± 4.1^a^	23.3 ± 3.6^b^	23.7 ± 3.6^a^	3.3 ± 0.3^a^
FT	16.3 ± 4.1^b^	9.3 ± 3.6^a^	7.0 ± 3.7^b^	2.5 ± 0.3^a^

*Values are mean ± standard error of the mean*.

a, b *Within columns, different letters between rows indicate significant differences (p < 0.05)*.

### Fresh Sperm Ultrastructure

**Figure 2:**
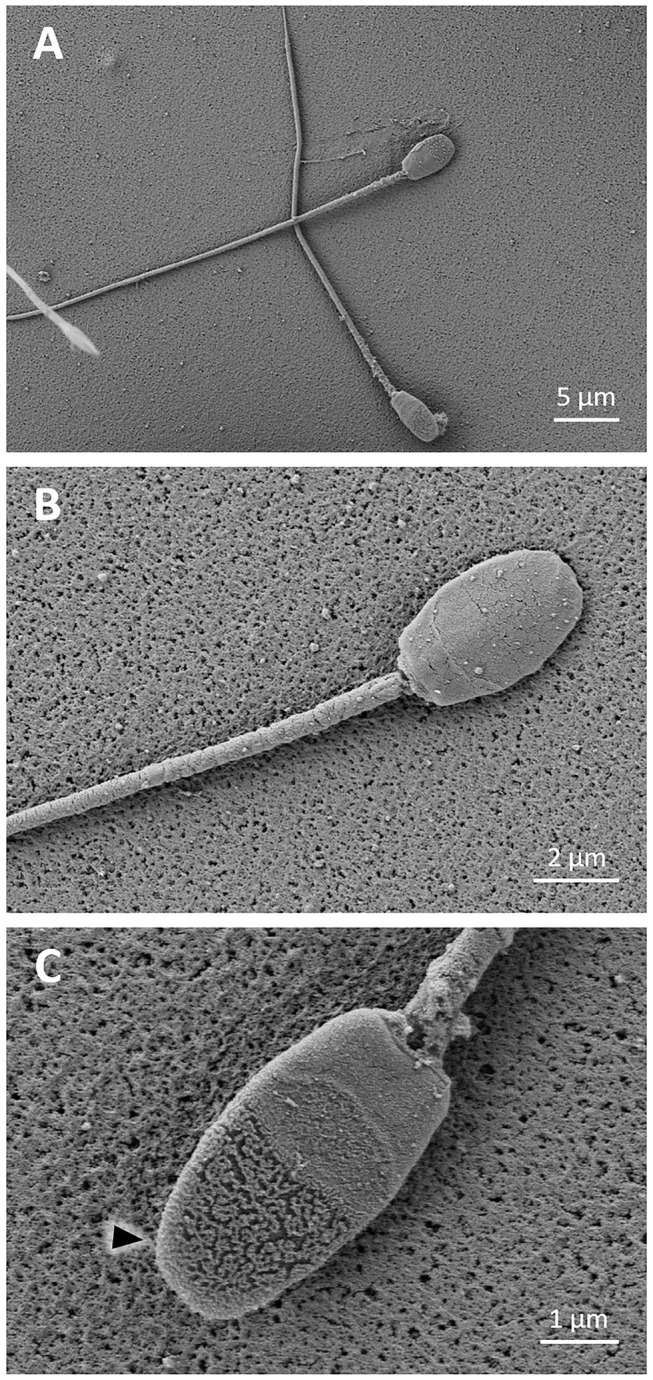
SEM images of fresh llama sperm. **(A)** Typical llama sperm morphology. **(B)** Sperm showing a slightly rough or cracked surface; the anterior acrosomal region, the equatorial segment, and the post-acrosomal region can be distinguished in the sperm head. **(C)** Sperm showing a typical acrosome reaction (triangle).

Prior to cryopreservation, sperm cells showed the typical morphology of llama spermatozoa (Figure 2A). Three well-differentiated regions could be detected in the sperm head: the anterior acrosomal region, the equatorial segment or posterior acrosomal region, and the post-acrosomal region (Figures 2B,C). While the anterior acrosomal region could be distinguished from the posterior acrosomal region by a straight line, the post-acrosomal region was virtually separated by a curved line. Lack of acrosomes was observed in some cells as a result of a premature acrosome reaction, revealed by the disruption of the plasma membrane in the anterior acrosomal region, whereas the post-acrosomal segment and its plasma membrane were preserved (Figure 2C).

**Figure 3:**
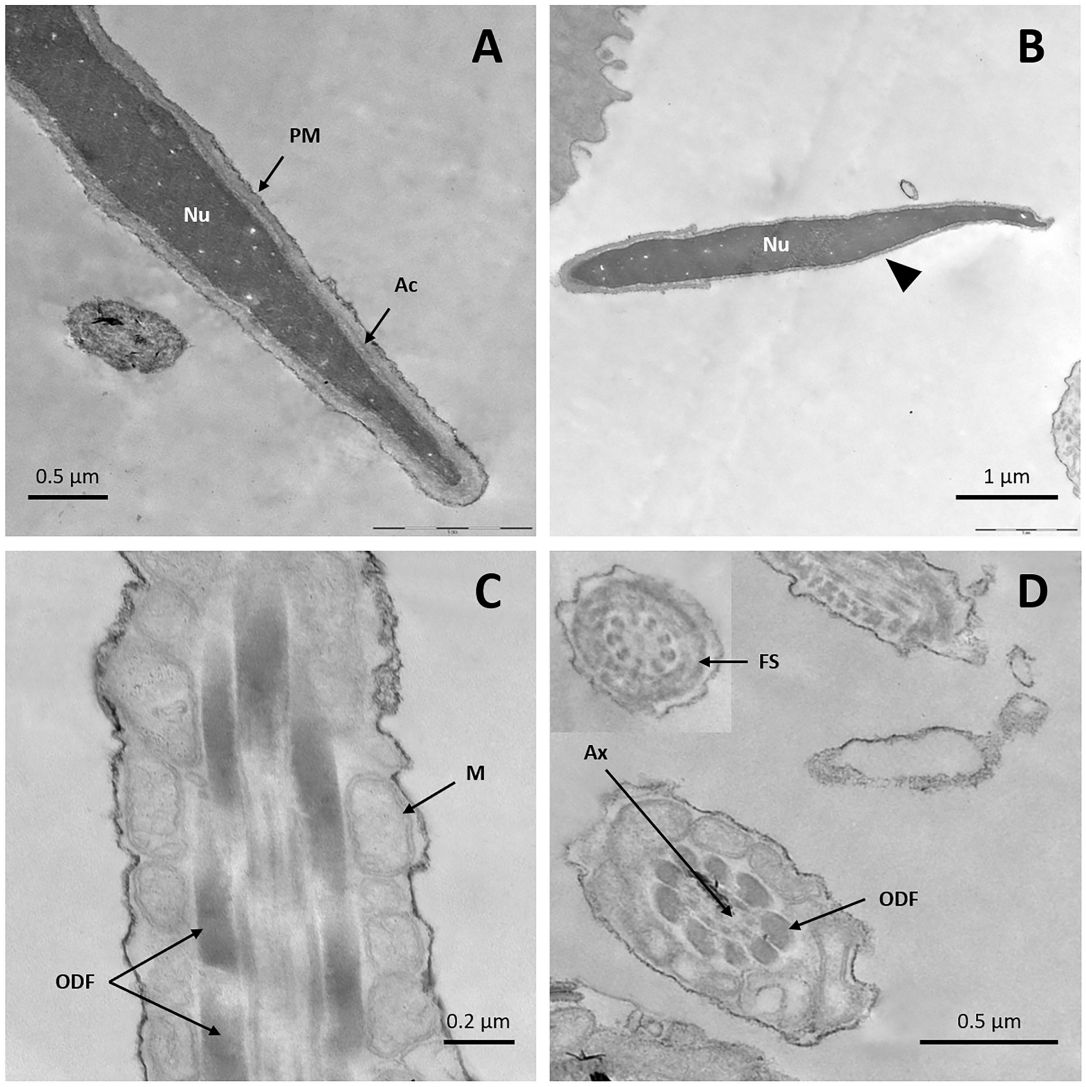
TEM images of fresh llama sperm. **(A)** Longitudinal section of the spermatozoon head showing an intact nucleus, acrosome, and plasma membrane. **(B)** Longitudinal section of the sperm head showing an intact nucleus and reacted acrosome (triangle). **(C)** Longitudinal section of sperm midpiece showing an intact plasma membrane and mitochondrial sheath. **(D)** Cross-section of the sperm tail showing the axoneme, the outer dense fibers and the fibrous sheath. Ac, acrosome; Ax, axoneme; FS, fibrous sheath; M, mitochondria; Nu, nucleus; ODF, outer dense fibers; PM, plasma membrane.

TEM micrographs of fresh sperm showed intact plasma membranes with homogeneous condensed chromatin (Figure 3A); only a tiny number of cells showed slightly undulated membranes in the sperm head. Acrosomal membranes and their content were preserved in most cells (Figure 3A), with only a few reacted sperm cells (Figure 3B). Well-preserved midpieces with the characteristic mitochondrial sheath surrounded by the plasma membrane were observed (Figure 3C). In the principal and mid pieces of the tail, presence of an intact axoneme with conventional structure was identified (Figure 3D), as well as presence of the typical nine outer dense fibers with drop formats around the axoneme (Figure 3D), and an external fibrous sheath (inset Figure 3D).

### Effect of Cooling on the Sperm Ultrastructure

**Figure 4:**
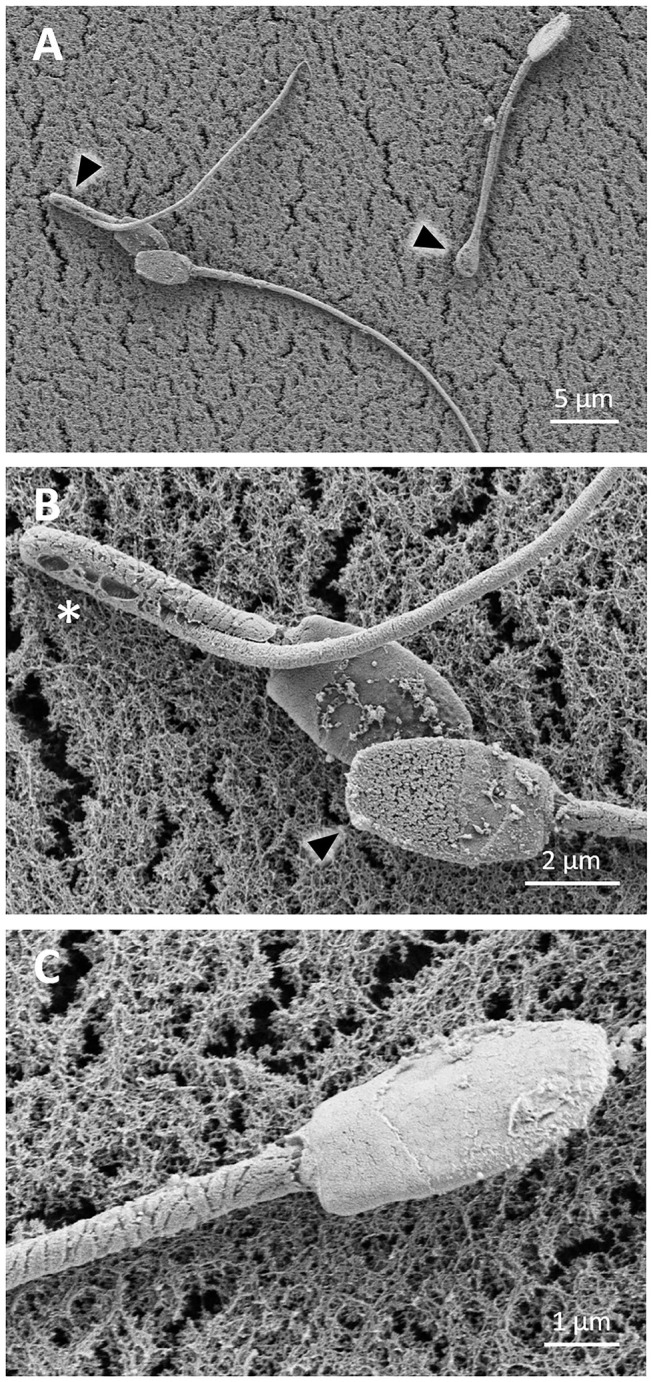
SEM images of llama sperm cooled for 24 h. **(A)** Sperm showing bent tails (triangle). **(B)** Sperm showing rough surface, bent tail (asterisk) and acrosome reaction (triangle). **(C)** Sperm showing cracked surface at the head level and loss of the plasma membrane in the midpiece.

Sperm showed signs of damage after 24 h of cooling. Some sperm cells showed bent tails (Figures 4A,B), with a rough or cracked surface, and other cells even showed loss of the plasma membrane (Figure 4C). Sperm cells with reacted acrosomes were also observed (Figure 4B).

**Figure 5:**
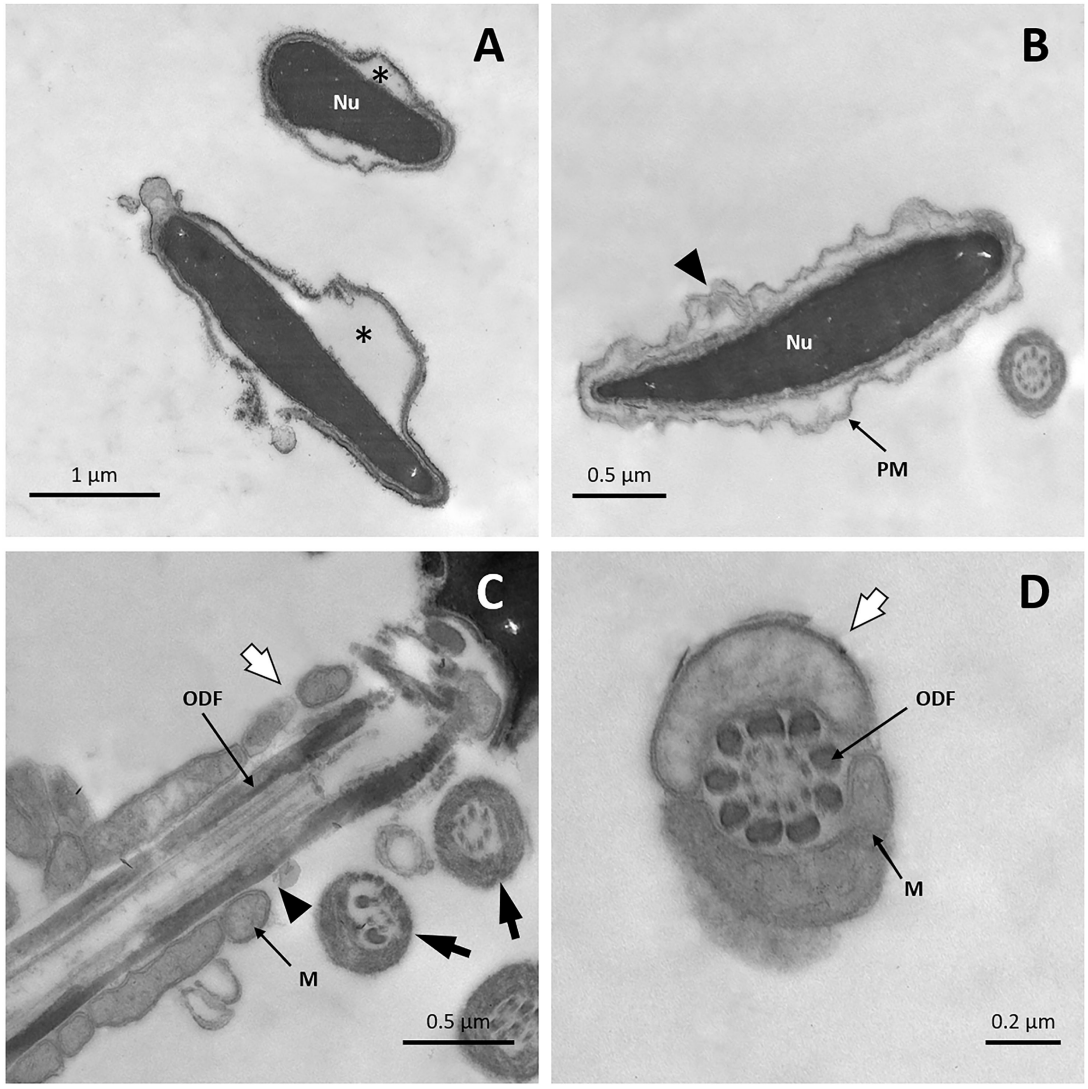
TEM images of llama sperm after 24 h of cooling. **(A)** Spermatozoa showing plasma membrane and acrosome detachment (asterisk). **(B)** Section of the spermatozoon head showing an irregularly undulated plasma membrane (triangle). **(C)** Longitudinal section of a sperm midpiece showing loss of the plasma membrane (white arrow) and mitochondria (triangle). Cross-sections of sperm tails with disorganized outer dense fibers and microtubules (black arrow). **(D)** Cross-section of the midpiece of a sperm cell showing an entirely loss of the plasma membrane (white arrow). M, mitochondria; Nu, nucleus; ODF, outer dense fibers; PM, plasma membrane.

TEM images showed many sperm cells with a swollen or irregular undulated plasmalemma in the head region, and a lack of acrosomal content (Figures 5A,B). In the midpiece, loss of the plasma membrane and mitochondria was detected (Figures 5C,D). Some alteration in the axoneme and periaxonemal structures such as changes in the number and arrangement of the microtubule doublets, or abnormal size and position of the outer dense fibers was observed (Figure 5C).

### Effect of Freezing on the Sperm Ultrastructure

**Figure 6:**
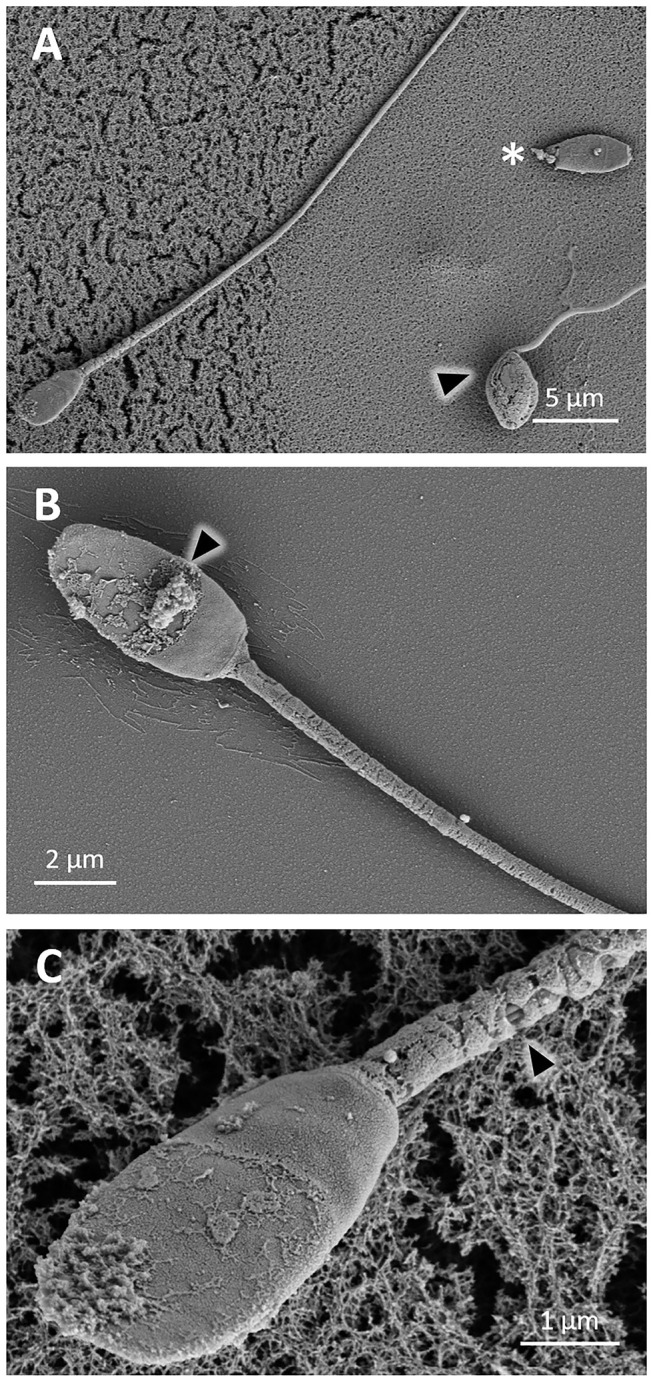
SEM images of llama sperm after cooling for 2.5 h. **(A)** Sperm cells showing detached head (asterisk) and coiled tail (triangle). **(B)** Sperm cell showing a rough surface (triangle). **(C)** Sperm cell showing loss of the plasma membrane and mitochondria (triangle) in the midpiece.

**Figure 7:**
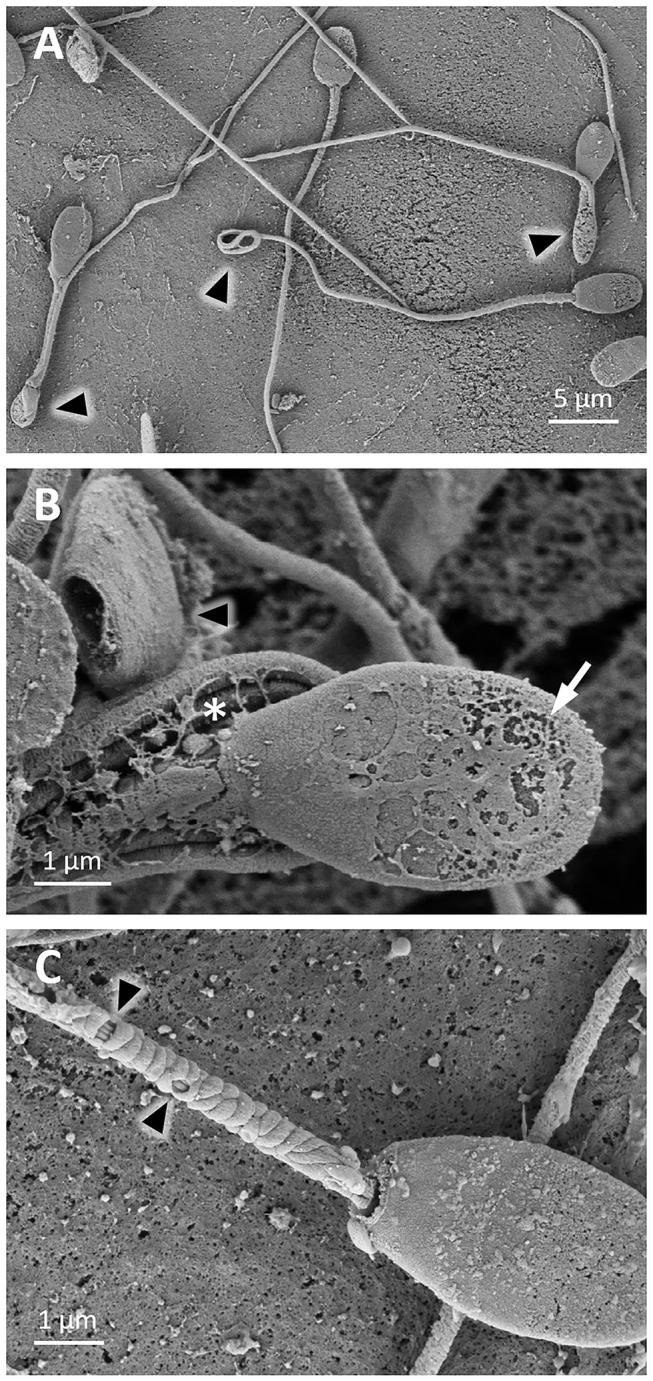
SEM images of llama sperm after thawing. **(A)** Sperm cells with bent and coiled tails (triangle). **(B)** Sperm cell with plasma membrane perforations (arrow) and coiled tail (asterisk), and a detached head (triangle). **(C)** Sperm cell showing loss of the plasma membrane and mitochondria (triangles) in the midpiece.

During the freezing procedure, sperm suffered cryodamage. After 2.5 h of cooling until 5°C, some detached heads and coiled tails were observed (Figure 6A). Sperm showed an irregular surface (Figure 6B), and broken membranes and loss of mitochondria were detected in sperm midpieces (Figure 6C). Similarly, after thawing, sperm showed detached heads as well as bent and coiled tails (Figures 7A,B). In the sperm head, the plasma membrane showed perforations (Figure 7B). In the midpieces, loss of the membrane and mitochondria could be distinguished (Figure 7C).

**Figure 8:**
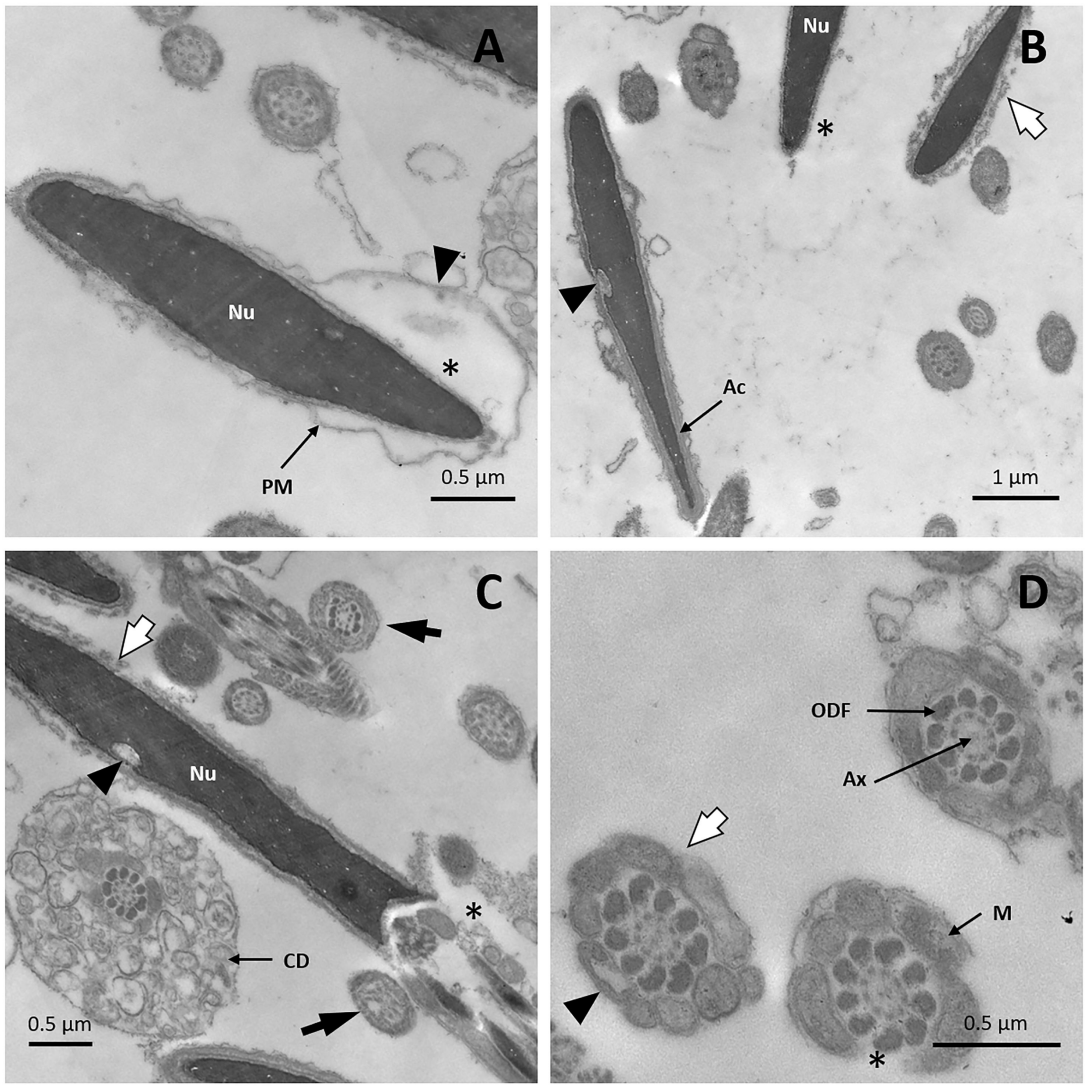
TEM images of llama sperm after 2.5 h of cooling. **(A)** Spermatozoon showing plasma membrane detachment (triangle) and a lack of acrosomal content (asterisk). **(B)** Sperm cells showing invagination in the nucleus (triangle) and acrosome reaction: it can be noticed that the outer acrosomal membrane and the plasma membrane form bubbles (white arrow), and that the acrosome is completely absent (asterisk). **(C)** Spermatozoon showing invagination in the nucleus (triangle), acrosome-reaction (white arrow), and loss of the plasma membrane in the midpiece (asterisk). Axonemal microtubule disorganization is also detected (black arrow). **(D)** Cross-section of the midpiece of a sperm cell showing the entire loss of the plasma membrane (white arrow). Missing (asterisk) and vacuolated (triangle) mitochondria are also observed. Ax, axoneme; CD, cytoplasmic droplet; M, mitochondria; Nu, nucleus; ODF, outer dense fibers; PM, plasma membrane.

After sperm cells were cooled until 5°C, but prior to freezing with liquid nitrogen, TEM images revealed detachment of the plasma membrane in many sperm heads (Figure 8A). Sperm without acrosome and with vesicles of fused plasma and outer acrosomal membranes was also observed, features that indicate the acrosome reaction (Figures 8B,C). In addition, some sperm cells displayed vacuoles in the nucleus (Figures 8B,C). In the midpiece, broken plasma membranes, mitochondrial vacuolization and even loss of mitochondria were noticed (Figures 8C,D). In the tail, some sperm cells presented an abnormal microtubule pattern (Figure 8C).

**Figure 9:**
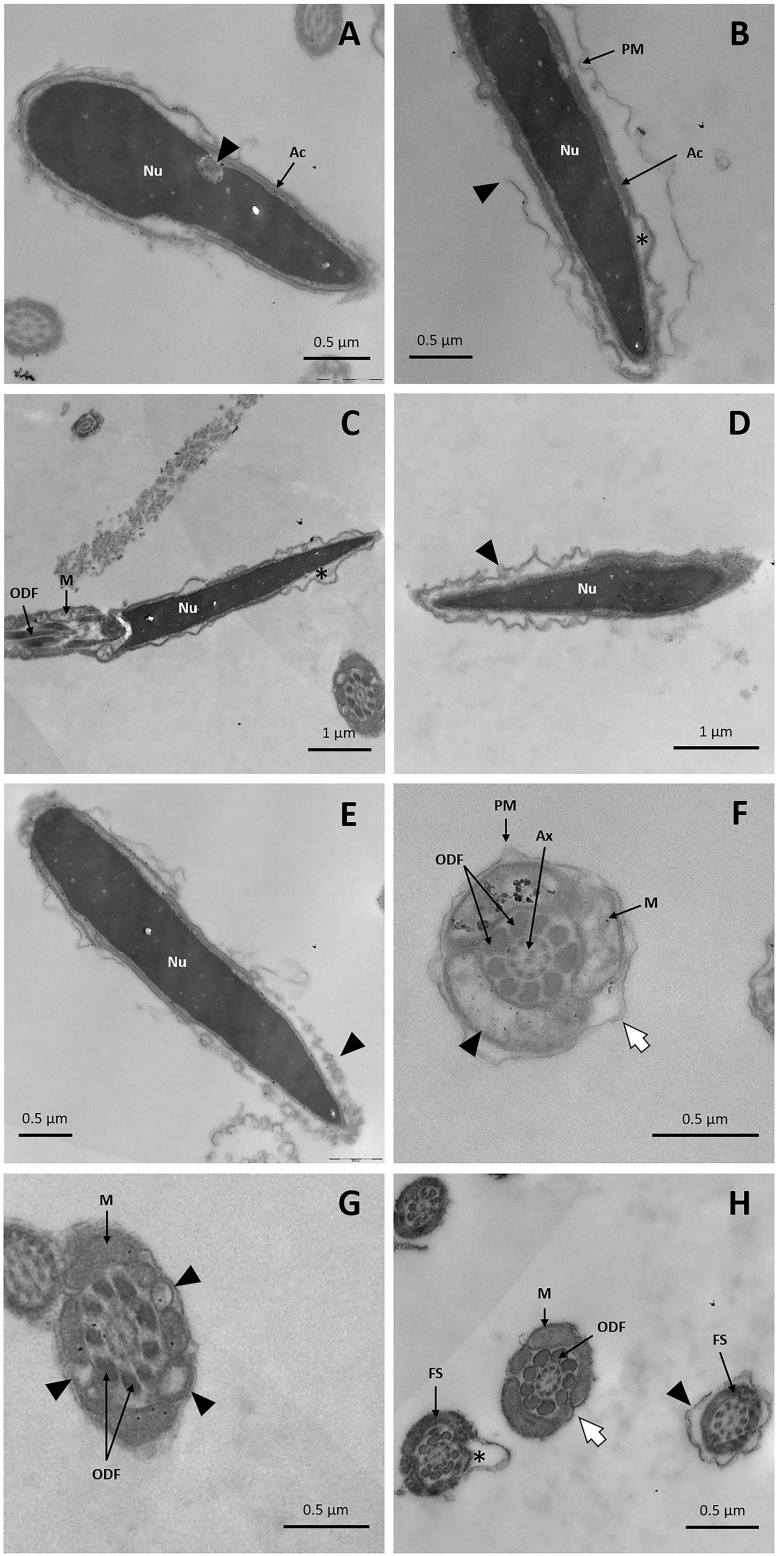
TEM images of llama sperm after thawing. **(A)** Presence of invagination (triangle) in the sperm nucleus. **(B)** Longitudinal section of the spermatozoon head showing an intact nucleus with broken plasma membrane (triangle) and a lack of acrosomal content (asterisk). **(C)** Longitudinal section of the sperm head and midpiece showing an irregularly undulated plasma membrane and the lack of acrosomal content (asterisk). **(D)** Sperm with swollen plasma membrane. **(E)** Acrosome-reacted spermatozoon: it can be noticed that the outer acrosomal membrane and the plasma membrane form bubbles (triangle). **(F)** Cross-section of the midpiece of a sperm tail showing plasma membrane detachment (white arrow) and mitochondria with distorted cristae (triangle). **(G)** Cross-section of a sperm midpiece showing mitochondria with vacuolization (triangles). **(H)** Cross-sections of sperm tails showing detachment of the plasma membrane in the principal piece (triangle), the entire loss of the plasma membrane in the midpiece (white arrow) and fibrous sheath disorganization (asterisk). Ac, acrosome; Ax, axoneme; FS, fibrous sheath; M, mitochondria; Nu, nucleus; ODF, outer dense fibers; PM, plasma membrane.

After freezing/thawing, great damage was detected with TEM. Examination of sperm cells revealed the presence of vacuoles in the sperm nucleus (Figure 9A). Acrosomal membranes appeared broken and there was a lack of acrosomal content (Figures 9B,C). Besides, certain cells exhibited the acrosome reaction with vesicles of fused plasma and outer acrosomal membranes (Figure 9E). The plasma membrane, particularly at the head level, was remarkably affected: spermatozoa were swollen, irregularly undulated, and demonstrated a broken plasma membrane (Figures 9B–D). Similar membrane alterations were detected in transverse sections of the midpiece and principal piece (Figures 9F–H). Examination of the sperm midpiece showed mitochondrial damaged: a distorted cristae structure and vacuolization (Figures 9F,G). The axoneme structure and outer dense fibers were conserved along the tail, but the fibrous sheath was damaged (Figure 9H).

## Discussion

Application of electron microscopy techniques to evaluate spermatozoa allowed detection of distressed cell structures after cryopreservation. In order to improve preservation procedures, llama sperm samples were first subjected to a cooling or freezing protocol and then evaluated using high-resolution microscopy techniques (SEM and TEM), to reveal any morphological injuries associated with cryopreservation methods by comparison with fresh sperm cells.

When llama spermatozoa were cooled until 5°C, sperm traits (viability, DNA integrity, motility, and vigor) were preserved after 24 of cooling. The presence of EY in the extender seems to be essential to maintain these sperm traits, as reported previously in llamas (13, 20). Even though cooling is not the most aggressive technique, SEM and TEM images revealed ultrastructural alterations affecting sperm quality after 24 h at 5°C. This might partly explain the poor results obtained when cryopreserved llama sperm is used in AI procedures (9–14). The ultrastructural changes observed would indicate that cooling protocols for llama semen need to be reviewed, evaluating different cooling rates, and diluents from an ultrastructural point of view.

Specifically, after 24 h of cooling, sperm showed signs of damage like cracked or loose membranes, reacted acrosomes, and bent and coiled tails. TEM observations also confirmed acrosomal damage, loss of mitochondria, and disorganization of the axoneme and periaxonemal structures. Several studies have demonstrated increased levels of reactive oxygen species (ROS) in semen preserved at 4°C (21–24). When the balance between ROS production and detoxification by antioxidants is disrupted, an excess of ROS creates oxidative stress. Consequences of oxidative damage are numerous, ranging from membrane damage and lipid peroxidation, inhibition of respiration, leakage of intracellular enzymes, axonemal protein damage, and mitochondrial membrane damage (25), some of which are in coincidence with our observations. The swollen and detached plasmalemma from the sperm head observed with TEM might also be associated with osmotic stress suffered during the cooling procedure, as has been seen in other species (26, 27).

During freezing, sperm traits were evaluated at the beginning of the process, after cooling until 5°C for 2.5 h, and after thawing. Similar to the cooling procedure, during which sperm cells were chilled for 24, after 2.5 h of cooling no differences were detected regarding sperm viability, DNA integrity, motility, and vigor compared with F0. As described for other species, the step from 5°C to liquid nitrogen seems to be the most critical one during freezing (4, 28), being sperm cells exposed to cold shock, ice crystal formation, and cellular dehydration, which severely compromise sperm quality (29).

Indeed, post-thawing sperm viability decreased from 66.9 ± 2.4% in F0 samples to 36.7 ±2.4% in frozen/thawed samples (~55% of sperm viability was retained with the protocol used in the present study). This is in agreement with previous reports on South American camelids, which described a similar decrease in sperm viability after thawing, even though different freezing protocols were employed (12, 30–32). With regard to sperm motility, a significant decrease in the percentage of total motility was observed after thawing when compared to F0 and F2.5. This decrease would be related to the decline in post-thaw viability. However, as reported in other studies, the impairment of mitochondrial activity (23, 33, 34), and axonemal protein damage (5) observed during freezing–thawing might also explain this result. On the other hand, DNA integrity would not be affected by the freezing procedure, and sperm cells also would be able to conserve their vigor with the protocol used in this study.

Although SEM and TEM images revealed similar alterations like those observed with the cooling protocol, sperm cryodamage seemed to be more severe after thawing. The plasma membrane surrounding the sperm head and tail were partially lost or exhibited various degrees of swelling, undulations, and perforations. Similar membrane injuries were detected after freezing-thawing in others species, e.g., bull (1), goat (15), dromedary (16), ram (17), and dog (18), demonstrating that the plasma membrane is one of the most sensitive parts of the sperm cell. Indeed, during freezing and thawing, the temperature, and osmotic variations induce tremendous alterations in cell water volume, which confer considerable mechanical stress on the cell membranes (7). Particularly, the swollen plasma membrane observed in all these species might be associated with osmotic shock suffered during freezing-thawing. It is clear that the plasma membrane exerts a fundamental role in the cell coating and cellular homeostasis, which means that membrane damage compromises the sperm function by altering the selective permeability of sperm membranes, and disrupting the ability of sperm to interact with cells of the oviduct and the oocyte (4, 7). In line with the above-mentioned issues, bull and buffalo sperm have shown a remarkable loss of capacity to regulate the intracellular concentrations of ions after cryopreservation (35, 36). Moreover, reports in several mammals have demonstrated that fewer cryopreserved sperm attached to the oviductal epithelial cells compared to fresh semen (37–39). Reduced sperm binding is likely a consequence of membrane injury, possibly by structural damage to the sperm receptors or by incomplete receptor aggregation (7).

After thawing, acrosomal integrity was seriously compromised. This became evident because of the absence of acrosomal content and acrosomal membrane damage observed with TEM. The acrosomal membrane is another sensitive part of the sperm cell to damage during cryopreservation (3). The loss of the acrosome has been associated with the mechanical stress suffered by the sperm during freezing or cryo-capacitation events that could lead to a premature acrosome reaction (7). In mammals, the acrosome reaction should occur in the vicinity of the mature cumulus-oocyte complex (40), and hence a premature acrosome reaction might result in reduced fertilization rates.

In the current study, presence of intranuclear vacuoles was observed after thawing, as has been detected in different species during semen cryopreservation (1, 15, 17). Even though vacuoles in the human sperm head were not associated with altered sperm traits or DNA damage (41), reports on different species have indicated that nuclear vacuoles are associated with abnormal chromatin packaging or chromatin damage (1, 42), and exert a negative effect on embryo development (43).

The mitochondrial structure was also affected by the freezing-thawing process. TEM and SEM images revealed vacuolization, cristae distortion and even loss of mitochondria in the midpiece of frozen sperm. Mitochondrial damage during freezing and thawing would lead to a decrease in spermatozoa mitochondrial membrane potential (MMP). Indeed cryopreservation (cooling and freezing) procedures significantly reduced the sperm MMP (23, 44, 45). Since high sperm MMP is required for mitochondrial ATP synthesis, the ability of the sperm cell to produce ATP could be compromised and therefore influence the sperm motility. Another sperm structure distorted by the freezing process was the fibrous sheath, which is a cytoskeletal structure surrounding the axoneme and outer dense fibers in the principal piece region of the sperm flagellum. The fibrous sheath acts as a scaffold for proteins, and is believed to influence the degree of flexibility, the plane of flagellar motion, and the shape of the flagellar beat (46). Other studies indicate that some cytoskeletal proteins of the tail decrease in abundance (e.g., ODF2, ROPN1, actin) or change their distribution during freezing–thawing in bull, ram, and buffalo sperm (47–50). These findings along with our observations might be associated with the loss of sperm motility during freezing-thawing.

Overall, the ultrastructural damage of llama spermatozoa caused by cryopreservation protocols appears to be irreversible, directly affecting sperm survival.

In conclusion, this is the first time that the ultrastructure of llama sperm and ultrastructural damage caused by cooling and freezing procedures have been characterized. Evaluations with SEM and TEM allowed us to detect several sperm ultrastructural injuries that would not be noticed by routine seminal assessments, and reveal alterations that start during the cooling procedure and intensify with freezing. In this context, further studies should be focused on a reduction in damage of the sperm plasma membrane, acrosome and mitochondria during cooling and freezing in order to improve cryopreservation protocols of llama semen.

## Data Availability Statement

The raw data supporting the conclusions of this article will be made available by the authors, without undue reservation.

## Ethics Statement

Protocol UNT 002/18 approved by the Committee for the Use and Care of Laboratory Animals (CICUAL) from Universidad Nacional de Tucumán.

## Author Contributions

RZ and SAA conceived and designed the study and interpreted the data. RZ, XAC-G, LMS, AM, AVD, MEA, and SAA collected samples and performed the experiments. RZ and LMS performed the statistical analysis. RZ wrote the draft manuscript. MEA and SAA revised and discussed the manuscript. All authors read and approved the manuscript for publication.

## Conflict of Interest

The authors declare that the research was conducted in the absence of any commercial or financial relationships that could be construed as a potential conflict of interest.

## References

[1] Khalil WA El-Harairy MA Zeidan AE Hassan MA Mohey-Elsaeed O. Evaluation of bull spermatozoa during and after cryopreservation: structural and ultrastructural insights. Int J Vet Sci Med. (2018) 6:S49–56. 10.1016/j.ijvsm.2017.11.001 30761321 PMC6161860

[2] Watson PF. The causes of reduced fertility with cryopreserved semen. Anim Reprod Sci. (2000) 60:481–92. 10.1016/S0378-4320(00)00099-3 10844218

[3] Pesch S Bergmann M. Structure of mammalian spermatozoa in respect to viability, fertility and cryopreservation. Micron. (2006) 37:597–612. 10.1016/j.micron.2006.02.006 16621580

[4] Ezzati M Shanehbandi D Hamdi K Rahbar S Pashaiasl M. Influence of cryopreservation on structure and function of mammalian spermatozoa: an overview. Cell Tissue Bank. (2020) 21:1–15. 10.1007/s10561-019-09797-0 31797118

[5] Peris-Frau P Soler AJ Iniesta-Cuerda M Martín-Maestro A Sánchez-Ajofrín I Medina-Chávez DA. Sperm cryodamage in ruminants: understanding the molecular changes induced by the cryopreservation process to optimize sperm quality. Int J Mol Sci. (2020) 21:2781. 10.3390/ijms21082781 32316334 PMC7215299

[6] Chantler E Abraham-Peskir JV. Significance of midpiece vesicles and functional integrity of the membranes of human spermatozoa after osmotic stress. Andrologia. (2004) 36:87–93. 10.1111/j.1439-0272.2004.00609.x 15084155

[7] Bailey JL Bilodeau JF Cormier N. Semen cryopreservation in domestic animals: a damaging and capacitating phenomenon. J Androl. (2000) 21:1–7. 10.1002/j.1939-4640.2000.tb03268.x 10670514

[8] Curry MR. Cryopreservation of semen from domestic livestock. Rev Reprod. (2000) 5:46–52. 10.1530/revreprod/5.1.46 10711735

[9] Bravo PW Skidmore JA Zhao XX. Reproductive aspects and storage of semen in *Camelidae*. Anim Reprod Sci. (2000) 62:173–93. 10.1016/S0378-4320(00)00158-5 10924824

[10] Aller JF Rebuffi GE Cancino AK Alberio RH Influencia de la criopreservación sobre la motilidad, viabilidad y fertilidad de espermatozoides de llama (*Lama glama*). Arch Zootec. (2003) 52:15–23. Available online at: https://www.redalyc.org/articulo.oa?id=49519702

[11] Vaughan J Galloway D Hopkins D Artificial insemination in alpacas (*Lama pacos*). Kingston, ACT: RIRDC Rural Industries Research and Development Corporation (2003).

[12] Stuart CC Vaughan JL Kershaw CM De Graaf SP Bathgate R. Effect of diluent type, cryoprotectant concentration, storage method and freeze/thaw rates on the post-thaw quality and fertility of cryopreserved alpaca spermatozoa. Sci Rep. (2019) 9:12826. 10.1038/s41598-019-49203-z 31492923 PMC6731240

[13] Giuliano SM Chaves MG Trasorras VL Gambarotta M Neild D. Development of an artificial insemination protocol in llamas using cooled semen. Anim Reprod Sci. (2012) 131:204–10. 10.1016/j.anireprosci.2012.03.010 22503638

[14] García W Alarcón V Bravo PW Inseminación artificial de alpacas con semen refrigerado y con inclusión de dos tipos de yema de huevo. Rev Investig Vet Perú. (2017) 28:337–44. 10.15381/rivep.v28i2.13080

[15] Shi L Ren Y Zhou H Hou G Xun W Yue W. Effect of rapid freezing–thawing techniques on the sperm parameters and ultrastructure of Chinese Taihang black goat spermatozoa. Micron. (2014) 57:6–12. 10.1016/j.micron.2013.09.004 24268840

[16] Shahin MA Khalil WA Saadeldin IM Swelum AAA El-Harairy MA. Comparison between the effects of adding vitamins, trace elements, and nanoparticles to shotor extender on the cryopreservation of dromedary camel epididymal spermatozoa. Animals. (2020) 10:78. 10.3390/ani10010078 31906462 PMC7022978

[17] Arando A Delgado JV Arrebola FA León JM Alcalá CJ Pérez-Marín CC. Vitrification induces critical subcellular damages in ram spermatozoa. Cryobiology. (2019) 87:52–9. 10.1016/j.cryobiol.2019.02.005 30826334

[18] Cerdeira J Sánchez-Calabuig MJ Pérez-Gutiérrez JF Hijon M Castaño C Santiago-Moreno J. Cryopreservation effects on canine sperm morphometric variables and ultrastructure: comparison between vitrification and conventional freezing. Cryobiology. (2020) 95:164–70. 10.1016/j.cryobiol.2020.03.007 32229272

[19] Ozkavukcu S Erdemli E Isik A Oztuna D Karahuseyinoglu S. Effects of cryopreservation on sperm parameters and ultrastructural morphology of human spermatozoa. J Assist Reprod Genet. (2008) 25:403–11. 10.1007/s10815-008-9232-3 18704674 PMC2582121

[20] Carretero MI Giuliano SM Arraztoa CC Santa Cruz RC Fumuso FG Neild DM. Comparison of two cooling protocols for llama semen: with and without collagenase and seminal plasma in the medium. Andrologia. (2017) 49:e12691. 10.1111/and.12691 27561901

[21] Wang AW Zhang H Ikemoto I Anderson DJ Lioughlin KR. Reactive oxygen species generation by seminal cells during cryopreservation. Urology. (1997) 49:921–5. 10.1016/S0090-4295(97)00070-8 9187701

[22] Vishwanath R Shannon P. Storage of bovine semen in liquid and frozen state. Anim Reprod Sci. (2000) 62:23–53. 10.1016/S0378-4320(00)00153-6 10924819

[23] Kadirvel G Kumar S Kumaresan A. Lipid peroxidation, mitochondrial membrane potential and DNA integrity of spermatozoa in relation to intracellular reactive oxygen species in liquid and frozen-thawed buffalo semen. Anim Reprod Sci. (2009) 114:125–34. 10.1016/j.anireprosci.2008.10.002 19010614

[24] Evangelista-Vargas S Santiani A. Detection of intracellular reactive oxygen species (superoxide anion and hydrogen peroxide) and lipid peroxidation during cryopreservation of alpaca spermatozoa. Reprod Domest Anim. (2017) 52:819–24. 10.1111/rda.12984 28455949

[25] Aitken RJ Gordon E Harkiss D Twigg JP Milne P Jennings Z. Relative impact of oxidative stress on the functional competence and genomic integrity of human spermatozoa. Biol. Reprod. (1998) 59:1037–46. 10.1095/biolreprod59.5.1037 9780307

[26] González-Fernández L Morrell JM Peña FJ Macías-García B. Osmotic shock induces structural damage on equine spermatozoa plasmalemma and mitochondria. Theriogenology. (2012) 78:415–22. 10.1016/j.theriogenology.2012.02.021 22578615

[27] Sieme H Oldenhof H Wolkers WF. Sperm membrane behaviour during cooling and cryopreservation. Reprod Domest Anim. (2015) 50:20–6. 10.1111/rda.12594 26382025

[28] Neild DM Gadella BM Chaves MG Miragaya MH Colenbrander B Agüero A. Membrane changes during different stages of a freeze–thaw protocol for equine semen cryopreservation. Theriogenology. (2003) 59:1693–705. 10.1016/S0093-691X(02)01231-1 12566145

[29] Hammerstedt RH Graham JK Nolan JP. Cryopreservation of mammalian sperm: what we ask them to survive. J Androl. (1990) 11:73–88. 2179184

[30] Santiani A Huanca W Sapana R Huanca T Sepúlveda N Sánchez R. Effects on the quality of frozen-thawed alpaca (*Lama pacos*) semen using two different cryoprotectants and extenders. Asian J Androl. (2005) 7:303–9. 10.1111/j.1745-7262.2005.00021.x 16110359

[31] Santiani A Evangelista S Valdivia M Risopatrón J Sánchez R. Effect of the addition of two superoxide dismutase analogues (Tempo and Tempol) to alpaca semen extender for cryopreservation. Theriogenology. (2013) 79:842–6. 10.1016/j.theriogenology.2012.12.012 23375779

[32] Carretero MI Neild DM Ferrante A Caldevilla M Arraztoa CC Fumuso FG. Effect of cryoprotectant and equilibration temperature on cryopreservation of *Lama Glama* spermatozoa. Andrologia. (2015) 47:685–93. 10.1111/and.12319 25059904

[33] Yoon SJ Kwon WS Rahman MS Lee JS Pang MG. A novel approach to identifying physical markers of cryo-damage in bull spermatozoa. PLoS ONE. (2015) 10:e0126232. 10.1371/journal.pone.0126232 25938413 PMC4418755

[34] Kumar D Kumar P Singh P Yadav SP Yadav PS. Assessment of sperm damages during different stages of cryopreservation in water buffalo by fluorescent probes. Cytotechnology. (2016) 68:451–8. 10.1007/s10616-014-9798-9 25373338 PMC4846636

[35] Bailey JL Buhr MM The impact of cryopreservation on Ca2+ regulation by bovine spermatozoa. Can J Anim Sci. (1994) 74:45–51. 10.4141/cjas94-007

[36] Kadirvel G Kumar S Kumaresan A Kathiravan P. Capacitation status of fresh and frozen-thawed buffalo spermatozoa in relation to cholesterol level, membrane fluidity and intracellular calcium. Anim Reprod Sci. (2009) 116:244–53. 10.1016/j.anireprosci.2009.02.003 19261396

[37] Dobrinski I Thomas PG Ball PA. Cryopreservation reduced the ability of equine spermatozoa to attach to oviductal epithelial cells and zonae pellucidae *in vitro*. J Androl. (1995) 16:536–42. 8867602

[38] Goldman EE Ellington JE Foote RH. Reaction of fresh and frozen bull spermatozoa incubated with fresh and frozen bovine oviduct epithelial cells. Reprod Nutr Dev. (1998) 38:281–8. 10.1051/rnd:19980308 9698279

[39] Burgess CM Clutterbuck AL England GCW. The effect of cryopreservation on the capacitation status and epithelial cell attachment capability of dog spermatozoa. Vet J. (2012) 192:398–402. 10.1016/j.tvjl.2011.08.026 21958721

[40] Yanagimachi R. Mammalian sperm acrosome reaction: where does it begin before fertilization? Biol Reprod. (2011) 85:4–5. 10.1095/biolreprod.111.092601 21490244

[41] Fortunato A Boni R Leo R Nacchia G Liguori F Casale S. Vacuoles in sperm head are not associated with head morphology, DNA damage and reproductive success. Reprod Biomed Online. (2016) 32:154–61. 10.1016/j.rbmo.2015.10.009 26655650

[42] Franco JG Jr Mauri AL Petersen CG Massaro FC Silva LFI Felipe V. Large nuclear vacuoles are indicative of abnormal chromatin packaging in human spermatozoa. Int J Androl. (2012) 35:46–51. 10.1111/j.1365-2605.2011.01154.x 21535011

[43] Vanderzwalmen P Hiemer A Rubner P Bach M Neyer A Stecher A. Blastocyst development after sperm selection at high magnification is associated with size and number of nuclear vacuoles. Reprod Biomed Online. (2008) 17:617–27. 10.1016/S1472-6483(10)60308-2 18983745

[44] Martin G Sabido O Durand P Levy R. Cryopreservation induces an apoptosis-like mechanism in bull sperm. Biol Reprod. (2004) 71:28–37. 10.1095/biolreprod.103.024281 14973261

[45] Guthrie HD Welch GR. Determination of intracellular reactive oxygen species and high mitochondrial membrane potential in viable boar sperm using fluorescence activated flow cytometry. J Anim Sci. (2006) 84:2089–100. 10.2527/jas.2005-766 16864869

[46] Eddy EM Toshimori K O'Brien DA. Fibrous sheath of mammalian spermatozoa. Microsc Res Techniq. (2003) 61:103–15. 10.1002/jemt.10320 12672126

[47] Felipe-Pérez YE Valencia J de Lourdes Juárez-Mosqueda M Pescador N Roa-Espitia AL Hernández-González EO. Cytoskeletal proteins F-actin and β-dystrobrevin are altered by the cryopreservation process in bull sperm. Cryobiology. (2012) 64:103–9. 10.1016/j.cryobiol.2011.12.004 22209823

[48] He Y Wang K Zhao X Zhang Y Ma Y Hu J. Differential proteome association study of freeze-thaw damage in ram sperm. Cryobiology. (2016) 72:60–8. 10.1016/j.cryobiol.2015.11.003 26617253

[49] Naresh S. Effect of cooling (4 °C) and cryopreservation on cytoskeleton actin and protein tyrosine phosphorylation in buffalo spermatozoa. Cryobiology. (2016) 72:7–13. 10.1016/j.cryobiol.2015.12.004 26725212

[50] Yoon SJ Rahman MS Kwon WS Ryu DY Park YJ Pang MG. Proteomic identification of cryostress in epididymal spermatozoa. J Anim Sci Biotechnol. (2016) 7:67. 10.1186/s40104-016-0128-2 27895910 PMC5117493

